# Supplementary material for: On the possibility of a terahertz light emitting diode based on a dressed quantum well
Source: Sci Rep. 2019 Nov 8;9:16320. doi: 10.1038/s41598-019-52704-6 (PMC6841726; doi:10.1038/s41598-019-52704-6)
Supplement: Supplementary file 1 — Supplementary Information [file 41598_2019_52704_MOESM1_ESM.pdf]

# Supplementary Information for
# On the possibility of a terahertz light emitting diode based on a dressed quantum well

## Derivation of the potential inside the p-n junction:

Let us consider an intrinsic QW sandwiched between a p-n junction having doping concentrations $N_a$ and $N_d$ in the p and n sides, respectively. Under the full-depletion approximation[1] the gradient of the electric field, $E$, along the growth direction, $z$, can be expressed using the Gauss's law:

$$\begin{aligned}
\frac{dE}{dz} &= -\frac{eN_a}{\varepsilon}, \text{ for } -(z_p + l/2) \leq z \leq -l/2. \\
\frac{dE}{dz} &= \frac{e(N_h - N_e)}{l\varepsilon}, \text{ for } -l/2 \leq z \leq l/2. \\
\frac{dE}{dz} &= \frac{eN_d}{\varepsilon}, \text{ for } l/2 \leq z \leq z_n + l/2.
\end{aligned} \tag{S1}$$

Here $e$ is the electronic charge; $\varepsilon$ is the permittivity of the material; $z_p$ and $z_n$ are the widths of the depletion regions in the p and n side, respectively; $l$ is the thickness of the QW; $N_h$ and $N_e$ are the number of holes and electrons per unit area inside the QW. Integrating Eq. (S1) and applying the continuous boundary conditions at the junctions yield

$$\begin{aligned}
E(z) &= -\frac{eN_a}{\varepsilon}(z + z_p + l/2), \text{ for } -(z_p + l/2) \leq z \leq -l/2. \\
E(z) &= \frac{e(N_h - N_e)}{l\varepsilon}z - \frac{e(N_h - N_e)}{2\varepsilon} - \frac{eN_d z_n}{\varepsilon}, \text{ for } -l/2 \leq z \leq l/2. \\
E(z) &= \frac{eN_d z}{\varepsilon} - \frac{eN_d z}{\varepsilon}(z_n + l/2), \text{ for } l/2 \leq z \leq z_n + l/2.
\end{aligned} \tag{S2}$$

From Eq. (S2) the expression for the potential can be obtained using

$$\phi(z) = \int E(z)\, dz \tag{S3}$$

## Derivation of $N_{e(h)}$:

$$N_e = \int_{E_c^0}^{\infty} \rho(E) f(E)\, dE, \tag{S4}$$

where $\rho(E) = m_e/\pi\hbar^2$, is the number of electronic states per unit area inside the QW and $f(E) = 1/\left[1 + \exp\left(\frac{E-F_n}{k_B T}\right)\right]$, is the Fermi distribution function. Substituting these inside Eq. S4 yields

$$N_e = \frac{m_e}{\pi\hbar^2} k_B T \ln\left[1 + \exp\left(\frac{F_n - E_c^0}{k_B T}\right)\right]$$

Following similar steps the expression for $N_h$ can also be obtained.

## References


1. B. van Zeghbroeck, *Principles of Semiconductor Devices and Heterojunctions* (Prentice-Hall, Upper Saddle River, 2010).
